# Segmentation of visceral and subcutaneous adipose tissue in abdominal CT-datasets with and without contrast medium: Influence of iterative reconstruction on 2D- and 3D-segmentation

**DOI:** 10.1016/j.ejro.2025.100707

**Published:** 2025-11-17

**Authors:** Robin F. Gohmann, Fyn Kaiser, Batuhan Temiz, Sebastian Gottschling, Christian Krieghoff, Christian Lücke, Matthias Horn, Matthias Gutberlet

**Affiliations:** a Department of Diagnostic and Interventional Radiology, Heart Center Leipzig, Leipzig, Germany; b Medical Faculty, University of Leipzig, Leipzig, Germany; c Institute for Medical Informatics, Statistics and Epidemiology (IMISE), University of Leipzig, Leipzig, Germany

**Keywords:** Body composition, Segmentation, Adipose tissue, Iterative reconstruction, Computed tomography

## Abstract

**Purpose:**

Segmentation of visceral and subcutaneous adipose tissue in computed tomography (CT) datasets has shown much promise in research and for medical applications, e.g. for risk stratification and guiding therapies. This study evaluates the influence of iterative reconstruction (IR) and filtered back projection (FBP) techniques on 2D- and 3D-segmentation of adipose tissue in CT images with and without contrast medium.

**Methods:**

We retrospectively analyzed 31 patients to compare adipose tissue density and quantity between IR and FBP across different compartments and contrast phases. Segmentation was performed using a fixed threshold (-190 to -30 HU).

**Results:**

Significant differences were observed in 2D-segmentation, particularly for visceral adipose tissue in non-enhanced scans (-0.54 ± 1.4 HU; *p* = 0.04) and subcutaneous adipose tissue in venous scans (-0.48 ± 1.2 HU; *p* = 0.03). In 3D-segmentation, subcutaneous adipose tissue density in venous scans was also lower with IR compared to FBP (-0.67 ± 1.2 HU; *p* = 0.004).

**Conclusion:**

Adipose tissue segmentation between IR and FBP revealed minimal and only occasionally yields statistically significant differences in density and quantity across adipose tissue compartments and contrast phases. The observed differences were very small, casting doubt on their clinical relevance at the level of individual patients. However, even subtle systematic variations may warrant consideration in population-based studies or longitudinal research where methodological consistency is critical.

## Highlights

• Minimal (<1 HU) differences in adipose tissue density found between IR and FBP.
• Differences between IR and FBP occurred mainly at tissue interfaces.
• Variations were statistically significant but are likely negligible for individual patients.
• Contrast medium application did not significantly affect segmentation differences.

## 1 Introduction

The segmentation of visceral and subcutaneous adipose tissue in computed tomography (CT) datasets plays a crucial role in various research and medical applications. Adipose tissue quantification can be utilized for risk stratification in conditions such as coronary heart disease or prior to valve replacement [1], [2]. Furthermore, the segmentation of adipose tissue enables longitudinal monitoring to track diseases progression or therapy response and e.g., can provide valuable information in intensive care patients [3]. Body composition analysis, and especially the measurements of adipose tissue can aid in the diagnosis and monitoring of conditions such as heart failure, portal hypertension, inflammation, oncotic problems, and lymphedema [4], [5], [6], [7].

**Fig. 1:**
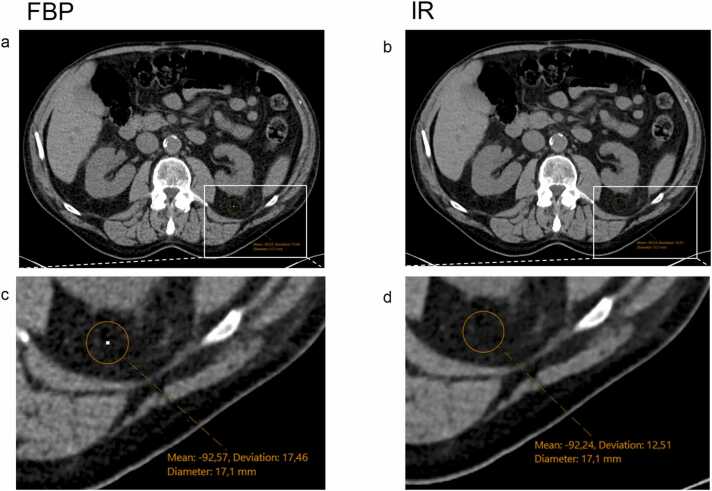
Exemplary CT slice (2D) of the abdomen at the L2/3 vertebral level without contrast medium, showing full abdominal views (a, b) and zoomed-in segments with density measurements (c, d). The images on the left were reconstructed using filtered back projection (FBP; a, c), the images on the right were reconstructed using iterative reconstruction (IR; b, d). Notably, the adipose tissue on the reconstructed with IR (right side, b and d) has a more homogeneous structure with lower density variations (lower noise). FBP=filtered back projection; IR=iterative reconstruction.

Various segmentation techniques and other sources of bias for the quantification and characterization of adipose tissue have been explored. Deep learning automated segmentation, the choice between 2D- and 3D-methods, acquisition parameters, slice thickness of reconstruction, and the influence of contrast media have all received attention [1], [8], [9], [10], [11], [12], [13], [14]. However, the influence of the reconstruction algorithm affecting image sharpness and noise (Fig. 1) and its relevance for adipose tissue segmentation has not been mentioned thus far [15], [16]. Understanding the impact of the reconstruction algorithm on segmentation is not only crucial for image quality but may also introduce systematic bias, especially when comparing longitudinal scans or data acquired from different scanners. Addressing this may help to improve comparability across imaging protocols and improve the accuracy and robustness of results.

The filtered back projection (FBP) algorithm operates by back-projecting the data along each ray or detector path in the image space, resulting in an unfiltered back projection. This unfiltered back projection creates a blurry image, which is then improved through the application of a suitable filter [17]. This can result in high noise (Fig. 1a and c) and may exaggerate streak artifacts [18]. This issue can frequently be overcome by using iterative reconstruction (IR) algorithms. In IR, an initial estimate of the image is made, which is typically a low-resolution or blurry image. This initial estimate is then refined iteratively by comparing it to the acquired projection data. The algorithm iteratively updates this image estimate up to an image that meets defined criteria [19], generally resulting in a less noisy image (Fig. 1b and d).

IR provides several advantages over direct reconstruction methods like FBP, including enhanced image quality through the reduction of noise (Fig. 1c and d), artifacts, and other image distortions. However, iterative reconstruction requires more computational resources and time compared to FBP, making FBP a still frequently employed reconstruction algorithm of the past, being still progressively replaced by IR over the past two decades.

The above-mentioned differences could potentially influence threshold-based segmentation of tissues. A better understanding of the influence of IR and FBP on adipose tissue segmentation could potentially enhance the accuracy and efficiency of adipose tissue segmentation in CT datasets, thus supporting its clinical application in various medical domains. Moreover, understanding the impact of iterative reconstruction and the choice of segmentation method could guide the development of future segmentation algorithms and imaging techniques.

Finally, threshold-based segmentation approaches carry the risk of including non-adipose structures such as intestinal content or fluid collections, particularly in the visceral compartment. This issue will be addressed in the course of this study.

In this study, we investigate the influence of IR and FBP reconstruction techniques on 2D- and 3D-segmentation methods of visceral and subcutaneous adipose tissue in CT datasets with and without contrast medium.

## 2 Materials and methods

### 2.1 Study design

The study population and design have previously been described [12]. We retrospectively included consecutive CT-studies of the abdomen from 31 patients who underwent both a non-enhanced scan (NES), and at least one contrast-enhanced scan (CES). The CT-scans were performed using the same CT-scanner with identical scan parameters. No enteral contrast medium was applied. Inclusion criteria required constant scan parameters within each examination between NES and CES, and the same application and amount of iodinated contrast medium (ICM). No further selection took place. A total of 95 scans were included: 31 NES, 23 arterial scans (ART), 10 portal-venous scans (PVN), and 31 venous scans (VEN).

### 2.2 CT acquisition, image reconstruction and analysis

CT images were acquired with the same Scanner (Siemens SOMATOM Flash, Siemens Healthineers), using the same tube potential and current for each scan series within the respective examination. The reference tube potential was 120 kV with automatic tube potential selection enabled, and the reference tube current was 180 mAs with automatic tube current modulation. Contrast injection protocol and scan delays have been described in detail elsewhere [12].

Images were reconstructed identically for all cases with a slice thickness and increment of 3 mm and in two specific algorithms (IR: I30f; FBP: B30f; Fig. 1). The IR reconstruction algorithm was an advanced modeled iterative reconstruction (ADMIRE, Siemens Healthineers).

The segmentation process was performed as described previously, using a specialized open-source software (3D Slicer, v4.10.2, http://www.slicer.org/) [10], [12], [20]. A threshold-based 2D-segmentation was performed at the same representative slice at L2/3. 3D-segmentation comprised the whole abdomen. The segmentation thresholds for adipose tissue were set between −190 and −30 HU [21].

### 2.3 Statistical analysis

Continuous variables were described using mean and standard deviation. To assess intra-patient differences between FBP and IR algorithms, paired *t*-tests were calculated at a significance level of 5 %. In addition, 95 % confidence intervals were computed for the estimated mean differences. Normality of paired differences was evaluated by visual histogram inspection and confirmed using the Shapiro–Wilk test. No significant deviation from normality was observed (p > 0.05).

**Fig. 2:**
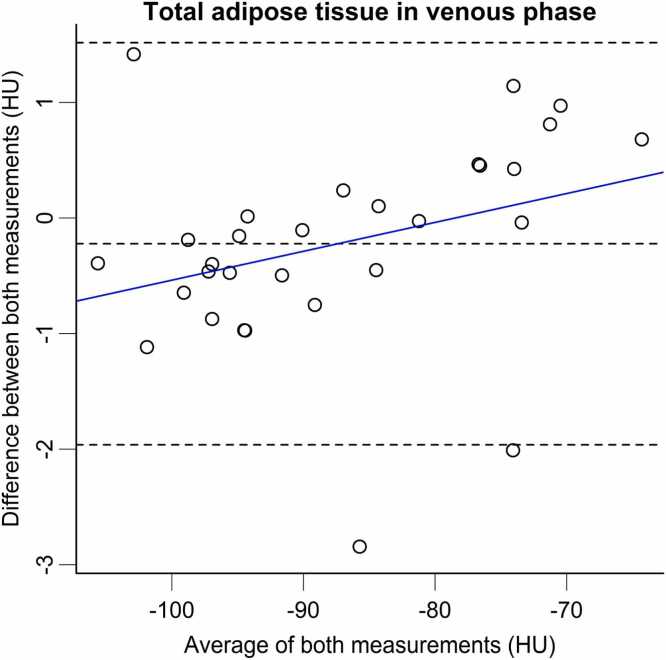
Bland-Altman plot illustrating the density in 3D of total adipose tissue in venous phase between FBP and IR. It demonstrates minor yet systematic differences between both methods (depicted by the solid blue linear regression line), all falling below 2 HU with the exception of one outlier. The mean difference and 95 % limits of agreement are represented by dashed lines. FBP=filtered back projection; HU= Hounsfield Units; IR=iterative reconstruction.

To examine the agreement between FBP and IR, Bland–Altman analysis was employed, wherein dashed lines show the mean difference between both methods as well as the 95 % limits of agreement, calculated as ±1.96 times the standard deviation of the differences (Fig. 2).

All statistical analyses were executed using R (v4.4.1, R Foundation for Statistical Computing, Vienna, Austria).

## 3 Results

### 3.1 Density

In our study, we observed small differences in density between IR and FBP in both 2D- and 3D-segmentation. In 2D-segmentation, significant differences were found in NES for visceral adipose tissue (VAT) (Δ −0.54 ± 1.4 HU; *p* = 0.04) and in VEN for subcutaneous adipose tissue (SAT) (Δ −0.48 ± 1.2 HU; *p* = 0.03). In 3D-segmentation, significant differences in density were noted in NES for total adipose tissue (TAT) (Δ −0.38 ± 0.9 HU; *p* = 0.03) and SAT (Δ −0.77 ± 1.5 HU; *p* = 0.006). Significant differences were also observed in the ART and VEN for SAT (Δ −0.86 ± 1.4 HU; *p* = 0.008; Δ −0.67 ± 1.2 HU; *p* = 0.004). Further details regarding the density of adipose tissue between IR and FBP are shown in Table 1.

**Table 1:** Differences in density of adipose tissue in non-enhanced and contrast-enhanced scans between iterative reconstruction and filtered back projection segmented in 2D and 3D-segmentation.

	**IR 2D (HU)**		**FBP 2D (HU)**		**Difference 2D (HU)**		***p***	**95 % CI (HU)**	**IR 3D (HU)**		**FBP 3D (HU)**		**Difference 3D (HU)**		***p***	**95 % CI (HU)**
**NES (n = 31)**																
TAT	−91.3	± 14.3	−91.0	± 14.1	−0.30	± 0.9	0.07	[−0.63, 0.02]	−90.2	± 12.5	−89.8	± 11.9	−0.38	± 0.9	**0.03**	[−0.71, −0.05]
VAT	−87.5	± 12.8	−87.0	± 12.7	−0.54	± 1.4	**0.04**	[−1.06, −0.02]	−84.1	± 12.7	−84.0	± 12.1	−0.03	± 1.3	0.89	[−0.50, 0.44]
SAT	−94.9	± 16.5	−94.7	± 16.2	−0.21	± 1.9	0.54	[−0.91, 0.49]	−94.7	± 13.2	−93.9	± 12.8	−0.77	± 1.5	**0.006**	[−1.30, −0.24]
**ART (n = 23)**																
TAT	−90.2	± 13.9	−90.2	± 13.1	0.07	± 1.7	0.85	[−0.65, 0.78]	−89.7	± 11.6	−89.3	± 11.1	−0.40	± 1.3	0.16	[−0.96, 0.17]
VAT	−86.0	± 12.6	−86.0	± 11.9	0.04	± 2.2	0.94	[−0.93, 1.00]	−83.5	± 12.0	−83.5	± 11.3	0.07	± 1.5	0.83	[−0.60, 0.74]
SAT	−94.7	± 15.0	−94.5	± 14.4	−0.18	± 1.6	0.60	[−0.89, 0.53]	−94.5	± 12.2	−93.6	± 11.8	−0.86	± 1.4	**0.008**	[−1,47, −0.25]
**PVN (n = 10)**																
TAT	−91.7	± 12.4	−91.4	± 11.8	−0.28	± 1.7	0.61	[−1.46, 0.91]	−91.0	± 10.0	−90.7	± 9.5	−0.32	± 0.8	0.26	[−0.92, 0.28]
VAT	−83.0	± 13.8	−81.6	± 10.5	−1.37	± 4.0	0.31	[−4.27, 1.52]	−79.7	± 11.2	−79.8	± 10.7	0.17	± 0.7	0.45	[−0.32, 0.66]
SAT	−97.6	± 11.9	−98.4	± 12.9	0.86	± 3.7	0.48	[−1.78, 3.50]	−98.0	± 9.9	−97.3	± 9.1	−0.72	± 1.2	0.09	[−1.55, 0.12]
**VEN (n = 31)**																
TAT	−87.6	± 14.0	−86.9	± 13.5	−0.21	± 1.1	0.28	[−0.61, 0.19]	−87.5	± 11.5	−86.8	± 11.4	−0.22	± 0.9	0.18	[−0.55, 0.11]
VAT	−82.3	± 13.3	−82.1	± 12.4	−0.13	± 1.4	0.61	[−0.66, 0.39]	−80.4	± 11.8	−80.2	± 11.1	−0.07	± 1.8	0.83	[−0.76, 0.61]
SAT	−92.3	± 15.4	−91.0	± 15.6	−0.48	± 1.2	**0.03**	[−0.92,- 0.05]	−92.6	± 12.5	−91.1	± 12.7	−0.67	± 1.2	**0.004**	[−1.11, −0.24]

Data are given in Hounsfield Units (HU); mean ± standard deviation. ART=arterial scans; CI=confidence interval; FBP=filtered back projection; IR=iterative reconstruction; NES=non-enhanced scan; PVN=portal-venous scans; SAT=subcutaneous adipose tissue; TAT=total adipose tissue; VAT=visceral adipose tissue; VEN=venous scans. The p value (and 95 % CI) corresponds to the change of density between FBP and IR. Significant p values are marked **bold**.

### 3.2 Quantity

There were minimal differences in adipose tissue quantity between IR and FBP in both 2D- and 3D-segmentation, without a clear directional tendency. Only the differences of segmented VAT in VEN segmented in 2D were statistically significant (0.03 ± 0.04 dm^2^, *p* < 0.001). The observed absolute mean differences in quantity with 2D-segmentation were below 0.03 dm². The absolute mean differences in adipose tissue segmented in 3D were below 0.06 dm³ with the exception of TAT in PVN and SAT in VEN (0.09 dm³ difference between IR and FBP, respectively). Further details regarding the area and volume of adipose tissue in IR and FBP are listed in Table 2.

**Table 2:** Differences in quantity of adipose tissue in non-enhanced and contrast-enhanced scans between iterative reconstruction and filtered back projection in 2D and 3D-segmented.

	**IR (dm**^2^)		**FBP (dm**^2^)		**Difference (dm**^2^)		***p***	**95 % CI (dm**^2^**)**	**IR (dm**^3^)		**FBP (dm**^3^)		**Difference (dm**^3^)		***p***	**95 % CI (dm**^3^**)**
**NES (n = 31)**																
TAT	3.94	± 1.54	3.92	± 1.52	0.02	± 0.07	0.11	[−0.004, 0.044]	10.69	± 4.94	10.68	± 4.84	0.02	± 0.29	0.76	[−0.091, 0.125]
VAT	1.92	± 0.91	1.93	± 0.92	−0.01	± 0.05	0.54	[−0.025, 0.013]	4.79	± 2.21	4.82	± 2.23	−0.02	± 0.14	0.35	[−0.075, 0.028]
SAT	2.02	± 0.96	2.00	± 0.94	0.03	± 0.07	0.06	[−0.001, 0.052]	5.90	± 3.44	5.86	± 3.31	0.04	± 0.26	0.40	[−0.056, 0.137]
**ART (n = 23)**																
TAT	4.08	± 1.50	4.08	± 1.50	0.01	± 0.07	0.76	[−0.027, 0.037]	10.89	± 5.12	10.85	± 5.13	0.04	± 0.30	0.57	[−0.092, 0.163]
VAT	2.03	± 0.91	2.01	± 0.91	0.02	± 0.05	0.10	[−0.003, 0.036]	4.86	± 2.21	4.85	± 2.22	0.01	± 0.11	0.60	[−0.035, 0.059]
SAT	2.06	± 0.98	2.07	± 0.99	−0.01	± 0.06	0.38	[−0.037, 0.015]	6.03	± 3.64	6.00	± 3.61	0.02	± 0.28	0.69	[−0.097, 0.144]
**PVN (n = 10)**																
TAT	3.91	± 1.65	3.91	± 1.65	0.00	± 0.03	0.90	[−0.020, 0.022]	11.01	± 5.02	10.92	± 4.97	0.09	± 0.17	0.14	[−0.035, 0.204]
VAT	1.70	± 0.94	1.69	± 0.92	0.01	± 0.03	0.21	[−0.010, 0.038]	4.30	± 2.09	4.26	± 2.14	0.04	± 0.13	0.41	[−0.059, 0.133]
SAT	2.21	± 0.94	2.22	± 0.95	−0.01	± 0.03	0.19	[−0.034, 0.008]	6.71	± 3.35	6.66	± 3.27	0.05	± 0.21	0.49	[−0.101, 0.196]
**VEN (n = 31)**																
TAT	3.77	± 1.58	3.73	± 1.53	0.03	± 0.09	0.05	[0.000, 0.063]	10.19	± 4.91	10.25	± 4.85	−0.06	± 0.24	0.20	[−0.147, 0.032]
VAT	1.78	± 0.95	1.77	± 0.92	0.03	± 0.04	**< 0.001**	[0.013, 0.044]	4.46	± 2.38	4.46	± 2.35	0.03	± 0.18	0.31	[−0.032, 0.099]
SAT	1.99	± 0.98	1.97	± 0.97	0.00	± 0.07	0.83	[−0.022, 0.028]	5.73	± 3.27	5.80	± 3.26	−0.09	± 0.25	0.06	[−0.184, 0.002]

Data are given in dm2 for area and dm3 for volume; mean ± standard deviation. ART=arterial scans; CI=confidence interval; FBP=filtered back projection; IR=iterative reconstruction; NES=non-enhanced scan; PVN=portal-venous scans; SAT=subcutaneous adipose tissue; TAT=total adipose tissue; VAT=visceral adipose tissue; VEN=venous scans; VOL=volume. The p value (and 95 % CI) corresponds to the change of area/volume between FBP and IR. Significant p values are marked **bold**.

### 3.3 Contrast medium

Several statistically significant differences in the density and quantity of segmented adipose tissue were observed between IR and FBP in NES and various contrast phases (ART, PV, VEN) (Table 1, Table 2). No significant differences were noted in PVN. The most noticeable differences in density and quantity were observed in NES and VEN, although no clear directional tendencies were evident.

## 4 Discussion

The use of IR instead of FBP changes the density and noise level in CT images and influenced quantity and density of segmented adipose tissue. The particular effect of the reconstruction algorithm on the segmentation was small and varied between NES and the different contrast phases and was more pronounced in 2D-segmentation than in 3D-segmentation. Therefore, the reconstruction algorithm may be a potential source of bias when comparing results to studies that used different reconstruction techniques. Even though these differences were small, they must not be overlooked entirely, particularly in longitudinal studies or risk stratification settings, where even subtle variations can affect clinical interpretation.

**Fig. 3:**
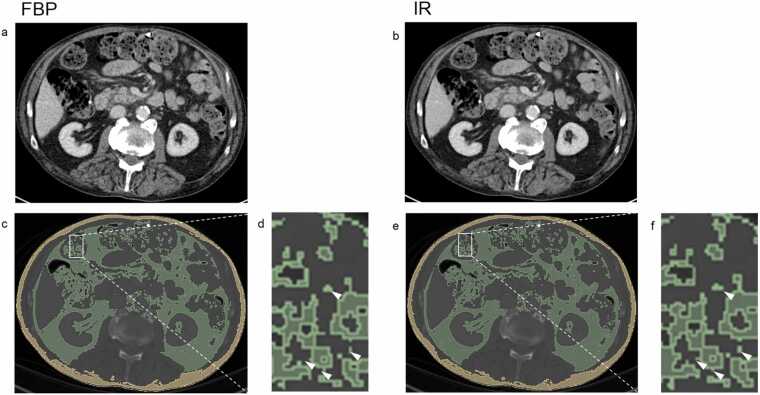
Exemplary CT slice (2D) of the abdomen at the level of L2/3 in venous contrast phase (a, b) and segmented adipose tissue of the same slice (c-f). The CT images on left were reconstructed using filtered back projection (FBP, a,c,d), the CT images on the right were reconstructed using iterative reconstruction (IR, b,e,f). Segmented visceral adipose tissue is marked in green, subcutaneous adipose tissue is marked in yellow. Minor differences in segmentation can be observed between the two reconstruction algorithms (IR and FBP) at tissue interfaces, e.g. in the vicinity of blood vessels or the bowel (arrowheads in d and f). FBP=filtered back projection; IR=iterative reconstruction.

The use of a different reconstruction algorithm alters the density by a small margin, most likely due to the classification of different voxels as adipose tissue, likely because of voxels with different density levels in close proximity and may also include partial volume effect (Fig. 1, Fig. 3). However, this effect is limited to the boundaries of different tissues and likely does not influence the majority of voxels classified within the set threshold. While the direction of density difference showed no clear trend, there were notable differences between 2D- and 3D-segmentation. 3D-segmentation yielded more statistically significant differences in density of segmented adipose tissue, probably due to the inclusion of more voxels in the volumetric analysis with neighboring voxels that had an influence on the voxels classified as adipose tissue, compared to the pixels in a single-slice image. Although the observed differences were below 1 HU and would be considered clinically negligible in routine imaging, emerging evidence suggests that even small shifts in adipose tissue density may hold prognostic value. For example, Pickhardt et al. recently demonstrated that differences in VAT density as small as 1 HU were independently associated with all-cause mortality in a large population-based cohort, especially among younger individuals and women [22].

Similar to our previous study that compared the segmentation of the entire abdomen with a single slice and examined the influence of contrast medium [10], we only observed small differences in adipose tissue quantity in both 2D- and 3D-segmentation. Overall, the reconstruction algorithm did not significantly influence segmented adipose tissue volume and led to a statistically significant difference in only one compartment in a specific contrast phase. The difference observed in VAT in venous-phase with 2D-segmentation can likely be explained by vascular enhancement affecting voxel density at fat–vessel interfaces. IR and FBP may classify these voxels differently due to noise behavior and partial volume effects (Fig. 3). This localized influence appears primarily in 2D-analysis, where a single slice is particularly sensitive to such regional variations, while 3D analysis is more robust. This suggests that the presence of vascular structures could affect the accuracy of segmentation, particularly in phases with more prominent vascular enhancement, albeit this difference was not observed in volumetric analysis, where such local variations are likely averaged out across the entire abdomen.

In the commonly used ICM phases, NES and VEN, slight differences in fat density between both reconstruction methods were noticeable primarily in SAT. These variations could be attributed to different factors, including the segmentation of small subcutaneous vessels. The boundaries between cutis and muscle tissues could have introduced additional challenges in adipose tissue segmentation, contributing to some degree of variability in the density measurements. Notably, PVN had fewer differences, but this observation might be due to a limited number of PVN scans available for analysis. Similar to the previous studies, the use and timing of ICM did influence the amount of segmented adipose tissue [10], [12]. But in contrast to the previous results, the application of contrast medium did not have a significant impact on the density, area, and volume of adipose tissue when comparing IR and FBP.

However, the threshold-based segmentation approach may inadvertently include non-adipose tissues or structures falling within the adipose tissue HU range (−190 to −30 HU), such as bowel contents. This can influence density and volume measurements, especially in 2D-analyses, where localized artifacts have a greater impact (sampling error). Future studies should address this limitation by implementing exclusion strategies such as anatomical masking, spatial constraints, or artificial intelligence to reduce misclassification and improve the reliability of adipose tissue quantification.

These findings correlate with other investigations, demonstrating less variability between FBP and IR reconstruction methods in lower density tissues like brain and other soft tissues while also improving contrast-to-noise- ratio [23], [24]. In tissues with larger density differences like bones [25], [26] or calcified coronary plaques higher differences have been reported [27], [28]. It is important to note that our observed differences in fat density were relatively small, typically less than 1 HU. While these differences were statistically significant, they may not be practically relevant in many clinical scenarios, falling within the margin of error. Nonetheless, small differences could propagate through clinical decision pathways, especially in follow-up or quantitative imaging applications, and should therefore be documented and controlled where possible.

Our findings indicate that the reconstruction algorithm applied generally does not have an impact on the measured amount of adipose tissue. The reconstruction algorithms utilized in our study lead to minimal differences in the quantity of segmented adipose tissue, whether in 2D- or 3D-segmentation, irrespective of the analyzed compartment or the acquired contrast phase with only one, likely coincidental significant difference in VAT in 2D-segmentation of the venous scan, which was likely caused by exaggerated tissue interfaces in the chosen slice, that could not be reproduced in other contrast phases or with 3D-segmentation. The results of the Bland-Altman-analysis indicate small, yet systematic differences between FBP and IR. These differences are subtle and likely too small to be clinically relevant (mean difference: −0.222 HU; 95 % limits of agreement: −1.962 and 1.518 HU; Fig. 2). However, this finding is even more important, as it opens the possibility to use these methods interchangeably. A larger sample size might be needed to prove these systematic differences statistically, but it is very unlikely, that these differences would become clinically relevant for decisions on patient level. Although the observed differences in measured adipose tissue amount were not statistically significant, it is essential to consider the possible clinical relevance of such subtle variations. Adipose tissue, especially VAT, has been shown to be relevant for risk stratification in various medical conditions. Prior studies have reported correlations between VAT amount and cardiovascular disease [29], [30], [31]. Furthermore, the amount of VAT has been linked to various metabolic parameters, as evidenced by associations with age, high triglyceride/low HDL levels, and BMI [32]. Small differences may influence the assigned risk or could potentially lead to misinterpretation during therapy monitoring and therefore affect a potential therapy. These correlations underscore the potential clinical significance of adipose tissue quantification in assessing the risk of metabolic diseases.

While it could theoretically be possible to transfer acquired data between FBP and IR using compartment- and contrast-phase–adjusted conversion formulas, the nature of the observed differences makes this impractical. The discrepancies are small and primarily arise at tissue interfaces, where differences in noise and reconstruction behavior affect voxel classification. As these effects vary across compartments and are influenced by local tissue characteristics rather than following a consistent pattern, applying generalized conversion formulas would likely introduce additional error. Although conversion formulas can aid in translating data between imaging techniques, their use in this context would require careful validation and compartment-specific adjustments, limiting their practicality.

Our findings might expand comparability between different scanning devices and could aid improving risk stratification and therapy monitoring. While the reconstruction algorithm has little practical influence on adipose tissue quantification at the level of individual patients, its subtle impact should be considered in standardized imaging protocols, especially in longitudinal or risk-sensitive contexts. This is increasingly relevant with the growing use of opportunistic screening, where previously acquired CT scans are repurposed for body composition analysis and may serve as baseline images for a newly acquired CT examination. The advent of fully automated, AI-based segmentation tools has further enabled large-scale, retrospective evaluation of adipose tissue across diverse populations and imaging protocols. In such settings, even small systematic differences introduced by varying reconstruction methods could confound pooled analyses or affect model calibration, and should therefore be acknowledged when interpreting or comparing results.

While our study employed ADMIRE, a model-based IR technique widely used in clinical CT, newer generations such as deep-learning–based iterative reconstruction (DLIR) may further modify image texture and voxel classification. As these algorithms build upon similar noise-reduction principles, we expect the general magnitude and direction of the observed effects to remain comparable. Nonetheless, validation in DLIR and other vendor-specific algorithms is warranted to confirm transferability of our findings.

### 4.1 Limitations

The retrospective study design may induce inherent biases and the sample size was relatively small, potentially limiting the generalizability of the results. Moreover, the unequal distribution of contrast phases, including the differing numbers of arterial phases, constrained the scope of subgroup analyses and comprehensive comparisons, especially for PVN.

The patient cohort exhibited varying underlying diseases and indications for examination, which could have affected the results. Moreover, subgroup analyses stratified by disease, gender or BMI could not be performed due to the limited sample size. However, the focus on intraindividual and intrascan comparisons aimed to mitigate systematic errors arising from these factors.

The use of only a single axial slice at L2/3 for 2D-analysis is a limitation. Future studies should consider including other anatomical levels to assess whether effects differ across regions. Moreover, subgroup analyses—such as for obese patients or individuals with ascites—could uncover more pronounced or compartment-specific effects and improve the generalizability of findings.

Furthermore, variations in patient size were present, despite applying the same amount of contrast medium. While this should not significantly alter the direction of change, its precise effect is unknown.

We did not perform a formal intra- or inter-observer variability analysis. However, due to the use of a fixed threshold segmentation protocol, direct intra-individual comparisons, and the clarity of anatomical compartments, along with initial training with the segmentation of 5 datasets multiple times and the generation of consistent data, the potential for observer-related variation was considered minimal.

All scans were acquired on a single scanner model from one vendor, and only one iterative reconstruction strength (IR=3) was evaluated. While this choice ensured standardized conditions, it may limit the generalizability of our findings to other vendors or different IR strengths. We selected IR= 3 because it provides a clinically acceptable balance between noise reduction and image texture, avoiding the minimal effect of IR= 1 and the overly smoothed appearance at IR= 5. Future studies should assess whether the observed effects are consistent across different vendors and reconstruction strength settings.

## 5 Conclusions

The comparison of segmentation of adipose tissue between IR and FBP reveals minimal and only occasionally yields statistically significant differences in density and quantity across adipose tissue compartments and contrast phases. The observed differences were very small, casting doubt on their clinical relevance at the level of individual patients. However, even subtle systematic variations may warrant consideration in population-based studies or longitudinal research where methodological consistency is critical.

## CRediT authorship contribution statement

**Christian Krieghoff:** Writing – review & editing, Investigation. **Matthias Horn:** Writing – review & editing, Formal analysis. **Christian Lücke:** Writing – review & editing, Investigation. **Matthias Gutberlet:** Writing – review & editing, Supervision, Resources, Project administration, Funding acquisition. **Fyn Kaiser:** Writing – review & editing, Writing – original draft, Visualization, Investigation, Data curation. **Gohmann Robin Fabian:** Writing – review & editing, Writing – original draft, Visualization, Software, Methodology, Investigation, Data curation, Conceptualization. **Sebastian Gottschling:** Writing – review & editing, Software, Methodology, Investigation. **Batuhan Temiz:** Writing – review & editing, Visualization, Investigation, Data curation.

## Informed consent statement

The ethics committee waived the requirement of written informed consent for participation from the participants or the participants’ legal guardians/next of kin because of the retrospective design and no identifiable data was being used.

## Institutional review board statement

The study was conducted according to the guidelines of the Declaration of Helsinki, and approved by the Ethics Committee of Leipzig University (reference number 337/19-ek).

## Funding

This research received no external funding. We acknowledge support from 10.13039/501100008678Leipzig University for Open Access Publishing.

## Declaration of Competing Interest

The authors declare no conflict of interest.

## Data Availability

The datasets generated and/or analyzed during the current study are not publicly available due to German Data Protection laws but are available from the corre-sponding author upon reasonable request after approval of the local ethics committee and data safety board.
